# Supplementary material for: Coupling the thermal acoustic modes of a bubble to an optomechanical sensor
Source: Microsyst Nanoeng. 2024 Dec 26;10:204. doi: 10.1038/s41378-024-00804-3 (PMC11669720; doi:10.1038/s41378-024-00804-3)
Supplement: Supplementary file 1 — Supplementary Material [file 41378_2024_804_MOESM1_ESM.docx]

**Supplementary Material: Coupling the thermal acoustic modes of a bubble to an optomechanical sensor**

K. G. Scheuer^1,2^, F. B. Romero^2^, and R. G. DeCorby^2^*

^1^Ultracoustics Technologies Ltd., Sherwood Park, AB, Canada, T8A 3H5

^2^ECE Department, University of Alberta, 9211-116 St. NW, Edmonton, AB, Canada, T6G 1H9

*Corresponding author: [rdecorby@ualberta.ca](mailto:rdecorby@ualberta.ca)

1. **Numerical Simulations**

Numerical simulations were performed using the ‘Pressure Acoustics’ module in COMSOL Multiphysics. The speed of sound in air and water were set to 340 m/s and 1500 m/s, along with densities of 1.29 kg/m^3^ and 997 kg/m^3^, respectively. Bubbles were modelled by surrounding a sphere of radius *R*_1_ (air) by a sphere of radius *R*_2_ (water). A perfectly matched layer of thickness *R*_1_ was assigned to the larger sphere, effectively creating a bubble surrounded by an infinite pool of water. A spherical wave radiation condition was applied to all exterior boundaries of the simulation. In the case of the tethered bubbles, a hard sound boundary (which sets the normal component of the acceleration to zero) was used to model the bubble/substrate interface, while the spherical wave radiation condition was kept for the bubble/water interfaces. A frequency-domain solver was used to extract the eigenmodes and the acoustic pressure distribution inside and around the bubble/water interface. For the tethered bubbles, the acoustic pressure distributions shown in the main text were taken at the bubble/substrate interface and at an orthogonal plane bisecting the bubble.

1. **Buckled-dome microcavities**

The microcavity sensors employed in this study are plano-concave optical resonators with 3.5 period Si/SiO_2_ Bragg mirrors deposited via plasma-enhanced chemical vapor deposition (PECVD). The cavities are realized using a thin-film buckling process, where an upper mirror is induced to buckle away from a lower mirror over circular regions of a patterned low-adhesion interface embedded between the mirrors [1]. Here, the resonant optical wavelength is nominally 1600 nm, such that the thicknesses of the Si (n = 3.57) and SiO_2_ (n = 1.46) layers were chosen as 112 and 274 nm, respectively. These materials were selected to provide sufficiently low optical loss over the wavelength range of interest, while also maintaining high index contrast. A typical optical scan is shown in Figure S.1(b), revealing a linewidth on the order of ~ 0.5 nm for the fundamental cavity mode.

The mechanical properties of the sensor are determined by the dimensions and material composition of the buckled top mirror (with a nominal total thickness of ~ 1270 nm and a base diameter of ~ 100 μm). The intrinsic vibrational properties of the buckled mirror was modeled using the solid mechanics module in COMSOL Multiphysics. The lowest-order predicted, radially symmetric vibrational modes are shown in Fig. S.1(a), alongside their corresponding resonant peaks in an experimental power spectral density plot measured in air. Optical read-out of mechanical signals is done by slightly detuning an interrogation laser from the fundamental optical resonance of the sensor (*i.e.,* the ‘tuned-to-slope’ technique), such that the motion of the concave upper mirror modulates the laser intensity, which is in turn read out using a high-speed photodetector receiver (see Section 5 below).

FIG. S.1. (a) Power spectral density plot showing mechanical resonances inherent to the sensor, captured in air. Corresponding modal profiles generated in COMSOL have been assigned to each centrosymmetric resonance. A grouping of five sensors is shown in the inset. Scale bar: 100 µm (b) Representative optical transmission measurement showing a family of modes, with the fundamental mode lying near 1592 nm. Corresponding modal profiles captured with an infrared camera have been assigned to each transmission peak. Reproduced from [1] with permission from the Royal Society of Chemistry.

1. **Analytical predictions for the Minnaert resonance frequency of tethered bubbles**

Most of the experimental observations described in this work were obtained for bubbles “tethered” to the surface of an optomechanical sensor chip. Numerous authors [2], [3], [4] have provided analytical predictions for a modified Minnaert breathing mode frequency in the case of a bubble tethered to a hard acoustic boundary. Notably, the breathing mode of a hemispherical tethered bubble is unchanged from that of a full spherical bubble of the same radius [2], [3], a result consistent with image theory at a hard boundary. For smaller contact angles (i.e., bubbles which lie between hemispherical and spherical), the Minnaert frequency at fixed *R* is reduced relative to the spherical case. The bubbles we studied typically had a contact angle of ~30 degrees, in which case *f*_TM_ ~ 0.83·*f*_M_ [3], where *f*_TM_ is the fundamental breathing mode of the tethered bubble. For the higher-order modes, experimental results were compared to COMSOL predictions.

1. **Estimated displacement associated with bubble modes**

The on-resonance displacement spectral density associated with a bubble acoustic mode can be estimated by applying the equipartition theorem to a linear harmonic oscillator model [4], which yields:

$S_{XX}(f_{n})\approx\frac{kTQ_{n}}{2\pi^{3}m_{n}f_{n}^{3}}$ . (S.1)

Here *f*_n_, *Q*_n_, and *m*_n_ are the resonant frequency, quality factor, and effective mass of the mode of interest.

As an example, consider the fundamental breathing mode of a spherical air bubble in water. Minnaert’s energy balance model [5] indirectly assigns an effective mass given by [6] *m*_n_ = 4π*ρR*^3^, where *ρ* is the density of water and *R* is the bubble radius. For the 100 μm radius bubble considered in Fig. 1 of the main text, for example, this yields *m*_n_ ~ 1.2×10^-8^ kg. Inserting this into Eq. S1 along with *f*_n_ ~ 33 kHz and *Q*_n_ ~ 50 [6] yields *S*_X_(*f*_n_) = {*S*_XX_(*f*_n_)}^1/2^ ~ 10^-13^ m/Hz^1/2^. Notably, this is several orders of magnitude higher than the displacement sensitivity of our optomechanical sensors (< 10^-16^ m/Hz^1/2^, see [7,8]).

A similar calculation can be carried out for the higher-order acoustic modes of the bubble, using a few approximations. First, since these high-order modes mainly involve air displacement, we let *m*_n_ ~ *m*_AIR_ (*i.e.*, the mass of the air inside the bubble). For the same spherical bubble above this yields *m*_n_ ~ 5×10^-12^ kg, and applying Eq. S1 with *f*_n_ ~ 1 MHz (a typical frequency for the first non-Minnaert mode) and *Q*_n_ ~ 50 yields *S*_X_(*f*_n_) ~ 10^-14^ m/Hz^1/2^, which is again well above the displacement-limited noise floor of our sensors.

Now, it is important to note that the coupling of the bubble displacements to the sensor displacement will depend in general on the overlap between their acoustic mode-field profiles. For example, the first non-Minnaert mode for the tethered bubbles has a pressure node at the location of the centrally aligned optomechanical sensor (see Figs. 3 and 4 of the main manuscript). Consistent with this, we typically observed a weaker signature for this mode in the sensor noise spectrum. Nevertheless, the approximate analysis above clearly suggests that it is entirely unsurprising that the bubble acoustic modes in general appear as strong features in the thermomechanical-noise-limited floor of our readout system.

1. **Experimental Setup and Instrumentation**

The optical measurement system was based on a reflection scheme similar to that described in previous work [1], [5], [6], and is shown schematically in Fig. S2. A tunable laser (Santec TSL-710) was tuned near the fundamental optical cavity resonance of a particular device and fiber-coupled into an optical circulator (Thorlabs RC08APC-P01) via SMF-28 fiber. The laser signal was then collimated and focused through an objective lens at the second port of the circulator. The free-space beam was aligned to the sensor/droplet using an infrared camera (Raptor Photonics Ninox 640). Light reflected from the device was returned back through the objective lens and collimator and into the optical circulator where it was fiber coupled directly into a high-speed, digitizing photodetector (Resolved Instruments DPD80).

Bubble dispensing was performed by first coating the 1 cm × 1 cm substrate with a thin layer of DI water held in place with surface tension. A syringe/needle combination was connected to a three-axis micro positioner and held over top of the substrate at a shallow angle. The needle was then brought into the water and a small volume of air was manually extruded, causing a tethered bubble to form at the needle tip. The micro positioner was then adjusted to bring the bubble into contact with the substrate before lifting the needle away. Figure S.2. shows a schematic view of the bubble sitting on top of a sensor. A supplementary video has also been included, showing how the position of a tethered bubble can be adjusted after the initial placement of the bubble on the substrate.


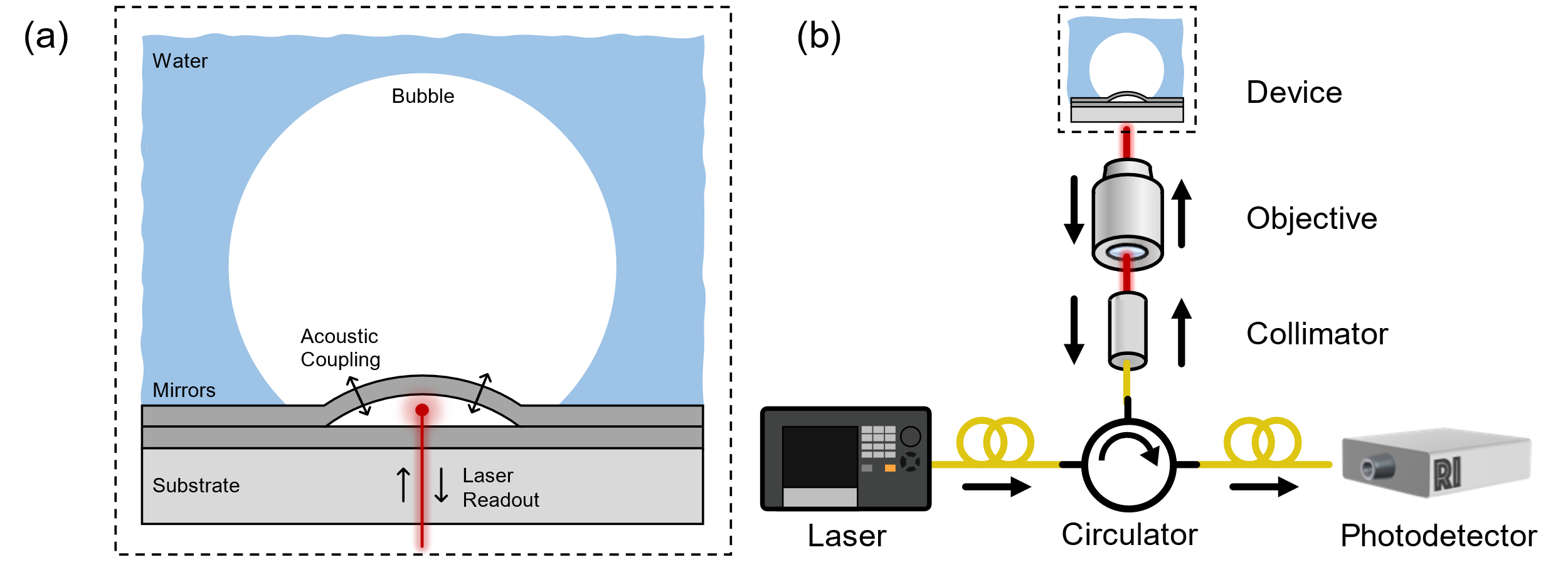


FIG. S.2. (a) Schematic cross-sectional view of a tethered bubble centered over a buckled dome Fabry-Perot optomechanical sensor. The motion of the flexible upper mirror is imprinted onto the laser light reflected from the optical cavity. The motion of the bubble is detected via the effect it has on the mirror motion. (b) Schematic of experimental measurement system. A tunable, fiber-coupled laser is directed into a circulator, collimated, and focused onto an optomechanical sensor through the back side of the substrate. The reflected laser signal is circulated into a photodetector.

1. **Data processing**

The Resolved Instruments (RI) detector samples received light at 80 Msamples/sec. For all of the plots shown in the main manuscript and below, the data is an average of the FFT algorithm applied to 300 individual time traces, each 10 msec in length (*i.e.*, 100 Hz resolution). In most cases, no further processing was applied. However, the ‘bulk’ air curves shown in Figs. 3-5 of the main manuscript were gently smoothed to show the trend line only while not obscuring the data for the sensor encapsulated by a bubble. The non-smoothed version of the bulk air curve can be seen in Fig. S.3 below. Moreover, the data shown in Figs. S.4 – S.7 below had smoothing applied to all curves.

1. **Quality factors for high-order acoustic modes of a tethered bubble**

The quality factors of the first 9 modes (excluding the Minnaert frequency) for the bubble shown in Fig. 4 of the main text were estimated and are tabulated in Table S.1.

**Table S.1. Quality factors of the resonances from Fig. 4 of the main text**

| Frequency (MHz) | Q factor |
| --- | --- |
| 0.39 | 28 |
| 0.64 | 42 |
| 0.77 | 44 |
| 0.88 | 46 |
| 0.99 | 35 |
| 1.15 | 74 |
| 1.22 | 37 |
| 1.45 | 60 |
| 1.65 | 45 |

1. **Supplemental data**

To augment the results presented in the main text, additional experimental data sets are shown below. Figure S.2 shows results for three different bubbles, in the same format as was shown for the bubble highlighted in Fig. 3 of the main manuscript. In each of these plots, the approximate location of the Minnaert resonance and of the first higher-order resonance are labeled.

FIG. S.3. Representative data, similar to that in Fig. 3 of the main manuscript, for three different bubbles. Here, the ‘bulk’ air noise spectrum is the gray line and the spectrum for the sensor encapsulated by the bubble is the red line. Neither curves are smoothed here. The upper left inset shows an overhead microscope image of the bubble in each case. For scale reference, the sensors (several are faintly visible around the bubble) are ~ 100 μm in diameter.

Figure S.4 shows two additional examples of normalized data, in the same format as provided in Fig. 4 of the main text. As in that case, the normalized power spectral density plots draw out the acoustic modes of the bubble with the weighting of the sensor’s own mechanical noise spectrum removed. In all cases, good alignment between COMSOL predictions and experimental observations was found for the first few modes. Some lower amplitude peaks are likely evidence that the bubbles do not have perfect cylindrical symmetry, so that nominally degenerate modes predicted in the symmetric case appear at slightly different frequencies in the experimental spectrum. At higher frequencies, the higher density of overlapping modes makes it more difficult to confidently assign features.


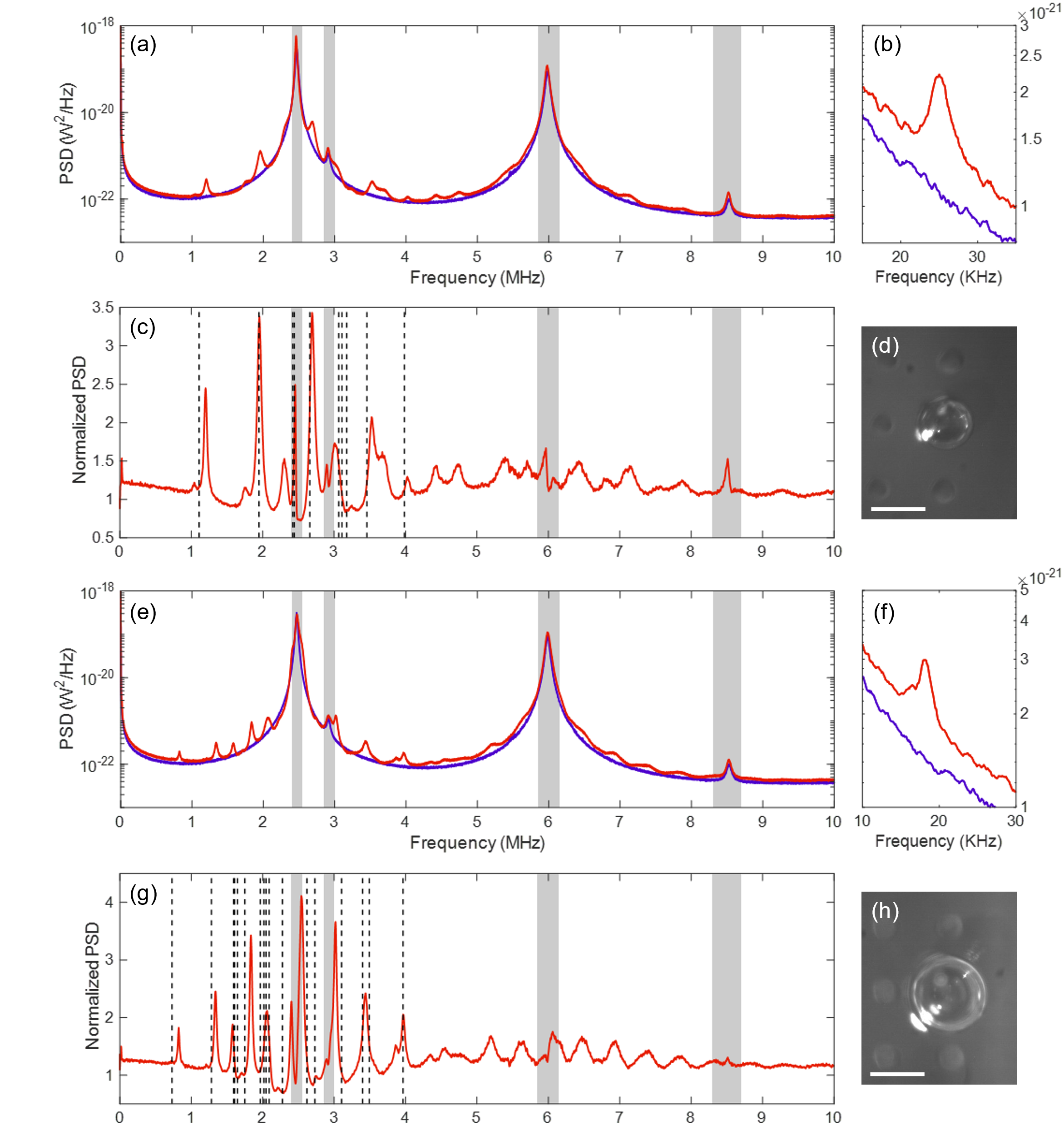


FIG. S.4. Additional bubble results with normalization applied. (a) Power spectral density for the bubble (red) referenced against a bulk air measurement (blue). (b) A zoomed region showing the Minnaert resonance. (c) A normalized power spectral density where the impact of the sensor was accounted for. The grey regions correspond to sensor resonances that may produce anomalous features in the normalized plots. The lower-amplitude peaks, such as at ~ 1 MHz, might be due to a slight bubble asymmetry which would lift mode degeneracy. (d) A top-down microscope image showing the bubble tethered on top of a sensor (Scale bar – 200 µm). (e-h) Analogous results for a slightly larger bubble (*note*: this is the same bubble shown in log-log form in Fig. 3 of the main manuscript). Dashed lines indicate numerical predictions from COMSOL.

1. **Detection of high-order vibrational modes for bubbles adjacent to a water-covered sensor**

Results in this section provide experimental evidence that the higher-order acoustic modes, as would be expected, exchange energy with the environment external to the bubble. To this end, we considered bubbles that were positioned near to an optomechanical sensor, but not centered overtop (and thus not encapsulating) the sensor as for the data presented above. The sensor is (mostly) covered in water in these cases, so that its own mechanical spectrum is different than in the air case [7].

First, we consider two cases where a larger tethered bubble ruptured and left behind a smaller bubble lying close to a sensor (see Fig. S.5). These accidental bubbles were smaller than those that could be created and placed with the needle/syringe. These bubbles were stable (and measured) over a period of several minutes. Note that it was not possible to estimate contact angles using a side-view image in these cases. However, it is well-known that smaller bubbles are more likely to assume a spherical shape, even next to a boundary.

FIG. S.5. Two examples of noise spectra captured for a sensor with a nearby, small bubble. Top-down microscope images are shown in the upper-left inset in each case. For scale reference, the sensor has a diameter of ~ 100 μm. From this, we estimated the bubble diameters were ~ 75 μm (left) and ~ 33 μm (right). In each case, a relatively low-quality (*Q* ~ 5) Minnaert resonance was observed, and its center frequency is labeled in the plot. For frequencies above ~ 0.8 MHz, a complicated spectrum was observed, and can be attributed in part to hybridization of the sensor and bubble acoustic modes. Note, for example, the Fano resonance and mode splitting features.

The power spectral density noise plots in both cases contained a clear signature of a Minnaert breathing mode resonance, and the center frequency of this feature is indicated in the plots. In each case, but especially for the smaller bubble, the observed resonance frequency is slightly lower than the expected Minnaert frequency (*f*_M_ ~ 3.3/*R* ~ 90 and ~ 200 kHz, respectively) using the radius estimated from the microscope images. This is consistent with the lowering of the Minnaert resonance frequency near a boundary, discussed above.

Above the fundamental dome resonance (at ~ 0.8 MHz in water [7]), the spectra (see Fig. S4) are quite complicated, and this makes it difficult to isolate features attributable to the higher-order bubble modes. Nevertheless, it is obvious that the vibrational spectrum of the dome is highly modified in the presence of the bubble. This could, in part, be due to an asymmetric ‘mass-loading’ effect, since the water pressure on the sensor mirror is not centro-symmetric in these cases. However, the data sets also contain classic features associated with coupled mechanical oscillators (*e.g.*, Fano resonances and mode splitting effects). This interaction is apparently quite complex, and a full treatment is left for future work. Nevertheless, the data hints that energy from the higher-order acoustic bubble modes is coupled to the external sensor, either through radiation into the water medium or via direct contact with the surface of the optomechanical device.

To further elucidate the external energy coupling, we performed a second set of experiments in which bubbles were suspended from a needle and held close to (but not in contact with) the optomechanical sensor. Figure S.6 shows a side-view image of a relatively large bubble tethered to a needle along with the measured power spectral density for the underlying sensor. Low amplitude “fringes” were observed when the bubble was positioned sufficiently close to the sensor. Also plotted are a series of grey lines corresponding to numerical predictions of the mode locations for an untethered spherical bubble on the same order of magnitude in size as the one shown in Fig. S.6(b). Discrepancies between the locations of the experimental peaks (some of which are indicated by the arrows) and the numerically predicted eigen-frequencies can be attributed to the non-spherical shape of this large bubble and the neglect of the chip boundary in the simulations. Given the weakness of the experimental features, it is also likely that some of the bubble resonances are lost in the noise floor of the sensor. Nevertheless, the data strongly hints that energy from the acoustic modes of the bubble reaches the underlying sensor via radiation through the water. Similar features were observed for multiple other combinations of suspended bubbles and sensors. It is worth reiterating that the bubble acoustic modes are driven only by room-temperature thermal Brownian motion in all of these experiments.

FIG. S.6. Evidence for detection of the acoustic modes of a bubble suspended from a needle. (a) Power spectral density plot of a tethered bubble held above, but not in contact with, a sensor. Low SNR features are present (black arrows) and their positions roughly align with the numerically predicted eigen-frequencies (grey dotted lines). (b) A side-view image of the bubble suspended over an optomechanical sensor. (Scale bar – 500 µm). (c) A normalized power spectral density plot more clearly showing the acoustic modes of the bubble. Note that modes were not assigned due to the comparatively poor signal-to-noise ratio.

1. **Further evidence of modified density of acoustic modes inside a small bubble**

To augment Fig. 6 in the main manuscript, Fig. S.7 shows a second small, well-centered bubble that exhibited evidence for Purcell-effect modification of the sensor’s vibrational energy. Insets highlighting the sensor’s mechanical modes at ~ 2.5 and ~ 6 MHz are plotted in Fig. S7(b-c). Similar to the data from Fig. 2 in the main text, there is strong evidence that the optomechanical sensor “sees” a modified acoustic density of modes when encapsulated by the small bubble. Specifically, note the suppression of background “emission” (*i.e.*, thermomechanical noise) over a wide frequency range except for enhancement at frequencies associated with the bubble acoustic modes, and the narrowing and red-shifting of the main sensor resonance due to a blue-detuned bubble mode just above 2.5 MHz (*i.e.*, see the slight ‘bump’on the blue side of the sensor’s Lorentzian lineshape).


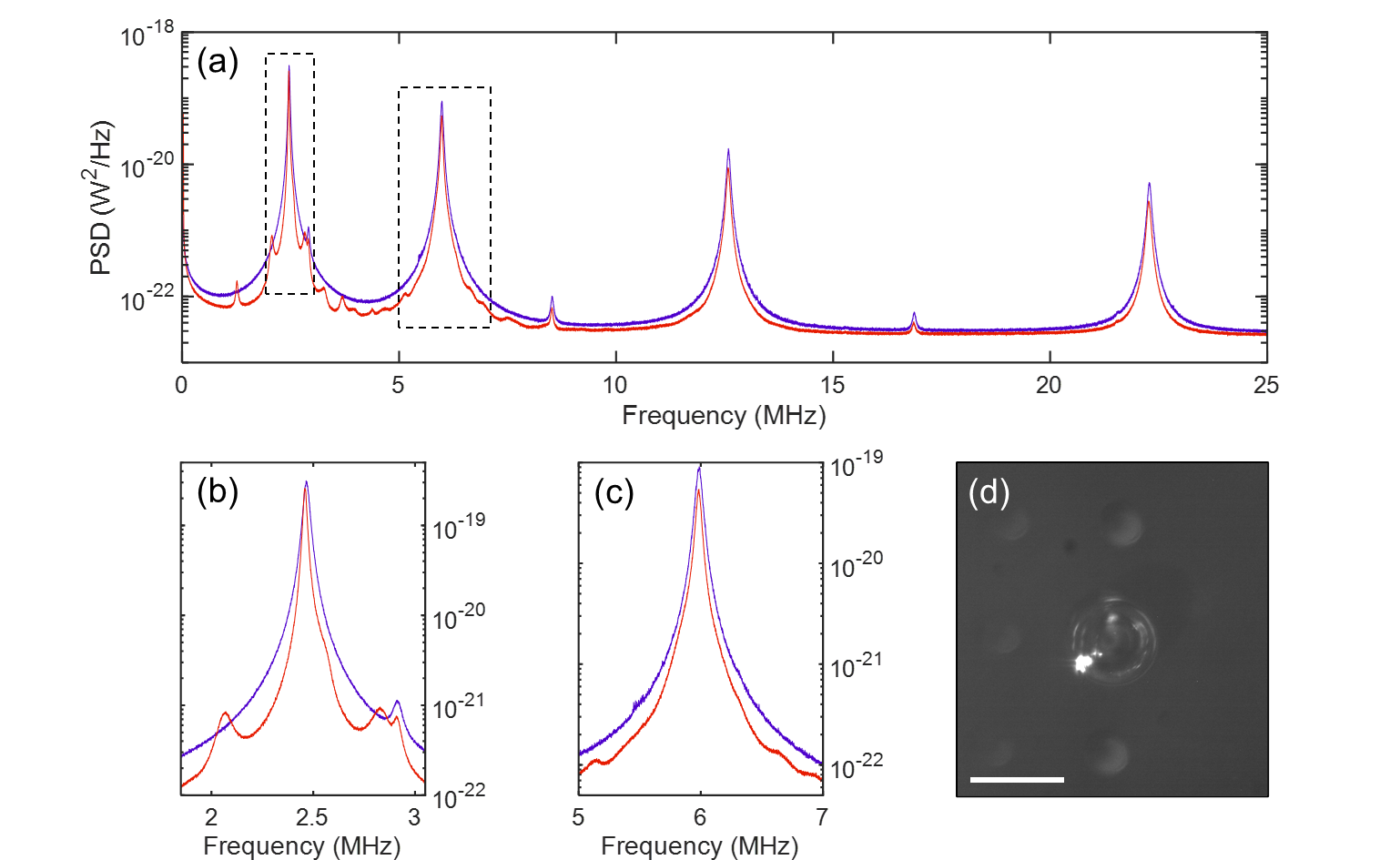


FIG. S.7. Additional evidence for the modification of the vibrational spectrum of an optomechanical sensor encapsulated by a small bubble. (a) Power spectral density plots for the same sensor, encapsulated by a small, well-centered bubble (red) and bulk air (blue) over the range 0 – 25 MHz. (b) A zoomed-in version showing the fundamental device resonance near 2.5 MHz. (c) A zoomed-in version showing the device resonance near 6 MHz. (d) A top-down microscope image showing the bubble tethered on top of a sensor (Scale bar – 200 µm).

**References:**

[1] K. G. Scheuer, F. B. Romero, G. J. Hornig, and R. G. DeCorby, “Ultrasonic spectroscopy of sessile droplets coupled to optomechanical sensors,” Jul. 13, 2023, *arXiv*: arXiv:2307.07009. Accessed: Aug. 15, 2023. [Online]. Available: http://arxiv.org/abs/2307.07009

[2] J. E. Blue, “Resonance of a Bubble on an Infinite Rigid Boundary,” *J. Acoust. Soc. Am.*, vol. 41, no. 2, pp. 369–372, Feb. 1967, doi: 10.1121/1.1910347.

[3] A. O. Maksimov, “On the volume oscillations of a tethered bubble,” *J. Sound Vib.*, vol. 283, no. 3–5, pp. 915–926, May 2005, doi: 10.1016/J.JSV.2004.05.021.

[4] R. Manasseh and A. Ooi, “Frequencies of acoustically interacting bubbles,” *Bubble Sci. Eng. Technol.*, vol. 1, no. 1–2, pp. 58–74, 2009, doi: 10.1179/175889709X446552.

[5] G. J. Hornig, K. G. Scheuer, E. B. Dew, R. Zemp, and R. G. DeCorby, “Ultrasound sensing at thermomechanical limits with optomechanical buckled-dome microcavities,” *Opt. Express Vol 30 Issue 18 Pp 33083-33096*, vol. 30, no. 18, pp. 33083–33096, Aug. 2022, doi: 10.1364/OE.463588.

[6] G. J. Hornig, K. G. Scheuer, and R. G. DeCorby, “Observation of thermal acoustic modes of a droplet coupled to an optomechanical sensor,” *Appl. Phys. Lett.*, vol. 123, no. 4, p. 42202, Jul. 2023, doi: 10.1063/5.0157924.
